# Specific Loss Power of Co/Li/Zn-Mixed Ferrite Powders for Magnetic Hyperthermia

**DOI:** 10.3390/s20072151

**Published:** 2020-04-10

**Authors:** Gabriele Barrera, Marco Coisson, Federica Celegato, Luca Martino, Priyanka Tiwari, Roshni Verma, Shashank N. Kane, Frédéric Mazaleyrat, Paola Tiberto

**Affiliations:** 1 Nanoscience and Materials Division, Istituto Nazionale di Ricerca Metrologica (INRiM), Strada delle Cacce 91, I-10135 Torino, Italy; m.coisson@inrim.it (M.C.); f.celegato@inrim.it (F.C.); l.martino@inrim.it (L.M.); p.tiberto@inrim.it (P.T.); 2 Magnetic Materials Laboratory, School of Physics, Devi Ahilya University, Khandwa road Campus, Indore 452001, India; priyanka.tiwari91092@gmail.com (P.T.); roshnikedar@gmail.com (R.V.); kane_sn@yahoo.com (S.N.K.); 3 Department of Physics, Prestige Institute of Engineering Management and Research, Indore 452010, India; 4 Laboratory of Systems & Applications of Information & Energy Technologies (SATIE), ENS University Paris-Saclay, CNRS 8029, 61 Av. du Pdt. Wilson, F-94230 Cachan, France; Frederic.MAZALEYRAT@ens-cachan.fr

**Keywords:** magnetic hyperthermia, specific loss power, magnetic mixed ferrites, hysteresis losses, thermometric measurements

## Abstract

An important research effort on the design of the magnetic particles is increasingly required to optimize the heat generation in biomedical applications, such as magnetic hyperthermia and heat-assisted drug release, considering the severe restrictions for the human body’s exposure to an alternating magnetic field. Magnetic nanoparticles, considered in a broad sense as passive sensors, show the ability to detect an alternating magnetic field and to transduce it into a localized increase of temperature. In this context, the high biocompatibility, easy synthesis procedure and easily tunable magnetic properties of ferrite powders make them ideal candidates. In particular, the tailoring of their chemical composition and cation distribution allows the control of their magnetic properties, tuning them towards the strict demands of these heat-assisted biomedical applications. In this work, Co_0.76_Zn_0.24_Fe_2_O_4,_ Li_0.375_Zn_0.25_Fe_2.375_O_4_ and ZnFe_2_O_4_ mixed-structure ferrite powders were synthesized in a ‘dry gel’ form by a sol-gel auto-combustion method. Their microstructural properties and cation distribution were obtained by X-ray diffraction characterization. Static and dynamic magnetic measurements were performed revealing the connection between the cation distribution and magnetic behavior. Particular attention was focused on the effect of Co^2+^ and Li^+^ ions on the magnetic properties at a magnetic field amplitude and the frequency values according to the practical demands of heat-assisted biomedical applications. In this context, the specific loss power (SLP) values were evaluated by ac-hysteresis losses and thermometric measurements at selected values of the dynamic magnetic fields.

## 1. Introduction

Nanotechnology addressed to a nanoscale design of materials is one of the utmost researched topics in the present century, involving disciplines like engineering, physics, chemistry and biology, concerning different application areas such as electronics, telecommunications, energy harvesting, sensors and biomedicine [1,2,3,4,5,6,7].

Although magnetic nanoparticles have been extensively studied in recent decades, they resulted in exciting materials to be used in these application areas due to their considerably size-dependent chemical and physical properties [8,9,10]. In particular, in the biomedical area, the tuning of the structure, size and composition of particles has led to the development of different applications such as magnetic biosensors, magnetic resonance imaging (MRI), drug-delivery and magnetic hyperthermia [6,11,12,13,14,15,16,17,18,19,20].

When exposed to an alternating magnetic field, magnetic particles, considered in a broad sense as passive sensors, are able to detect and transduce it in a controlled and localized release of heat; this ability has promoted the use of these materials for advanced therapeutic applications such as magnetic hyperthermia and heat-assisted drug release [13,21,22,23]. The physical mechanism at the base of heat generation has been recently identified to be mainly the magnetic hysteresis losses [24]. The efficiency of the heat generation, estimated by the specific loss power (SLP) value, depends on several parameters; some of these can be identified as “external”, such as the intensity and the frequency of the applied magnetic field and the liquid medium properties, whereas others, identified as “internal”, depend on the intrinsic properties of the magnetic particles such as composition, size, shape and magnetic state [25,26].

Because of the strict restrictions required on the applied alternating magnetic field parameters for the human body’s exposure [27,28,29], a huge research effort should be primarily focused on the design of the intrinsic properties of magnetic particles in order to optimize the efficiency of the heat release according to practical demands [30,31].

In this context, spinel ferrite powders attract extraordinary attention because of their high biocompatibility, easy synthesis procedure, physical and chemical stability and easily tunable magnetic properties [32,33,34,35,36,37]. The general formula of ferrite is MeFe_2_O_4_ where Me represents a divalent metal ion (e.g., Fe^2+^, Co^2+^, Ni^2+^, Zn^2+^, etc.) or a combination of ions with the average valence of two (e.g., Li^+^ and Fe^3+^ in lithium ferrite, etc.) [33]. Moreover, combinations of these ions are also possible, obtaining mixed-structure ferrites with different compositions [33,38].

Generally, in the biomedical area, ferrite particles based on magnetic Co^2+^ ions are proposed as promising heat generators due to their strong magnetocrystalline anisotropy and moderate magnetization [39,40,41]. Instead, Li^+^ ions, combined in magnetic (Li^+^_0.5_Fe^3+^_0.5_) species in ferrite structures, have attracted attention because of their low toxicity [42,43]. On the other hand, the non-magnetic Zn^2+^ ion is usually used as a partial substitutional of magnetic divalent ions or species in order to finely tune the magnetic properties of the ferrite particles [38], especially the saturation magnetization [33].

Together with the chemical composition changes, the manipulation of the cation distribution on octahedral and tetrahedral sites represents another suitable strategy to control the magnetic behavior of ferrites due to the strong connection between the spinel structure and its magnetism [44,45,46,47].

The biocompatibility evaluation of ferrite particles represents a preliminary and fundamental step towards their use in biomedical applications. The variety of ions in the ferrite composition represents one of the several parameters that influence the viability of the cells [48,49]; e.g., Co-ferrite is less biocompatible than Fe_3_O_4_ and Mn-ferrite [50]. However, different coating materials such as polymer or surfactants can be used as protective layers minimizing the direct exposure of the less biocompatible ions present on the ferrite surface to the biological environment [39,51].

In the present work, Co_0.76_Zn_0.24_Fe_2_O_4,_ Li_0.375_Zn_0.25_Fe_2.375_O_4_ and ZnFe_2_O_4_ mixed-structure ferrites were synthesized in a ‘dry gel’ form by a sol-gel auto-combustion method. Here, X-ray diffraction (XRD) was exploited to calculate the cation distribution. Static and dynamic magnetic characterizations were performed to study the connection between the cation distribution and magnetic behavior. Particular attention was devoted to clarifying the role of Co^2+^ and Li^+^ ions on the magnetic behavior of ferrites at a magnetic field amplitude and the frequency values appropriate to foresee heat-assisted biomedical applications. Moreover, the hysteresis losses and thermometric measurements at selected values of ac-magnetic fields were performed to evaluate the specific loss power (SLP) of the samples.

## 2. Materials and Methods

Li_0.375_Zn_0.25_Fe_2.375_O_4_, Co_0.76_Zn_0.24_Fe_2_O_4_ and ZnFe_2_O_4_ powder ferrite samples were synthesized by sol-gel auto-combustion method [52]. In summary, the powders were synthesized via utilizing AR grade citrate-nitrate/acetate precursors (Zn(NO_3_)_2_.6H_2_O, Ferric nitrate—Fe(NO_3_)_3_.9H_2_O, Lithium acetate—CH_3_.COOLi.2H_2_O, cobalt nitrate—Co(NO_3_)2 6H_2_O and citric acid—C_6_H_8_O_7_), were mixed in the stoichiometric ratio. Citric acid was used as the ‘fuel’ and the ratio of metal salts to fuel was taken as 1:1. In the beginning, the citric acid acted as a chelating agent for the metal ions of varying ionic sizes, which helped in preventing their selective precipitation to maintain compositional homogeneity among the constituents. Subsequently, it also served as a fuel in the combustion reaction. To start the synthesis process, all the precursor materials for the desired composition were dissolved in deionized water in a beaker under constant stirring, to get a homogeneous solution and the pH was maintained at 7 by adding ammonia solution. Then, the solution was heated at ~110 °C for 1 h in air till a fluffy powder was formed called ‘dry gel’ or ‘as-burnt powder’, which was ground to get a fine powder. After the synthesis, the Li_0.375_Zn_0.25_Fe_2.375_O_4_ and ZnFe_2_O_4_ samples were annealed at 450 °C for 3 h; instead, no post-synthesis heat treatments were performed on the Co_0.76_Zn_0.24_Fe_2_O_4_ sample.

The Zn concentration (x ≈ 0.25) and the resulting composition of LiZn- and CoZn-ferrites were chosen in order to induce an increase in the saturation magnetization compared to the corresponding simple ferrites (CoFeO and LiFeO) [33,53], taking into account the practical requests to optimize the heating efficiency. A higher concentration of Zn ions (x > 0.5) should be avoided as it lowers the magnetization saturation. [33,53].

The structural properties of powder samples were analyzed by X-ray diffraction (XRD) using Cu-K_α_ radiation (wavelength ‘*λ*’ = 0.1541 nm) in θ–2θ configuration (step size of 0.02°), equipped with a fast counting Bruker LynxEye detector, with Silicon strip technology. Rietveld refinement was performed by MAUD (material analysis using diffraction) software [54]. XRD data were analyzed to obtain structural parameters: experimental (*a_exp._*) lattice parameters, X-ray density (*ρ_xrd_*) and mean grain diameter (<*D_xrd_*>). The distribution of cations on tetrahedral and octahedral sites in the studied samples was determined by analyzing the XRD pattern, employing the Bertaut method [55], as was also reported in earlier reports [56,57,58]. XRD intensity depends on the atomic position in the spinel unit cell whereas the XRD peak position relies on the size and the shape of the unit cell. Bertaut’s method utilizes the following pair of reflections: (400), (422) and (220), (400), according to the expression:

(1) $$\frac{I_{hkl}^{obs}}{I_{h’k’l’}^{obs}}=\frac{I_{hkl}^{cal}}{I_{h’k’l’}^{cal}}$$

where *I_hkl_^obs^* and *I_hkl_^cal^* are respectively the observed and estimated intensities for the reflection (hkl). These ratios were evaluated for the numerous groupings of cationic distribution at tetrahedral and octahedral sites as described in [56]. The best distribution of cations was taken among the sites for which theoretical and experimental lattice parameters agreed clearly.

Room-temperature-static-magnetization curves were measured by the means of a vibrating sample magnetometer (VSM), operating in the magnetic field range −1200 < *H* < 1200 kA/m. The dc-hysteresis loops were measured at selected vertex fields in the interval 0–1200 kA/m. From the major loops, the coercive field (*H_c_*), the magnetic remanence (*μ_0_M_r_*) and the saturation magnetization (*μ_0_M_s_*) were evaluated; specifically, the latter was determined by fitting the high-field portion of dc-hysteresis curves with the standard expression $\mu_{0}M=\mu_{0}M_{s}-a/H$, representing the first-order approximation of the series expansion that describes the law of approach to saturation [59].

Room-temperature dynamic hysteresis loops were measured by the means of a custom-built B-H loop tracer [60] operating with the ac-magnetic field amplitude in the range 8–42 kA/m at the fixed frequency of ~69 kHz; the ac-hysteresis loops were measured at selected vertex fields in the allowable range. The area enclosed by ac-hysteresis loops was calculated in order to estimate the specific loss power by the means of the hysteresis losses (SLP).

Thermometric measurements were performed by an ad hoc-developed hyperthermia setup described in detail elsewhere [60]. In summary, the magnetic particle suspension at desired concentration was placed in the center of a copper coil with a diameter of about 5 cm which generated an electromagnetic field with a frequency of 100 kHz and an intensity up to 47.7 kA/m. The geometry of the set-up guaranteed a homogenous field through the entire sample and the used field frequency and intensity fell within the general safety [27,28]. The magnetic particle suspension was prepared by dispersing the Li_0.375_Zn_0.25_Fe_2.375_O_4_ powder in a liquid medium, consisting of sodium citrate tribasic dispersed in deionized water (0.2 g/L), obtaining a magnetic solution concentration of about 27 mg/mL. Sodium citrate tribasic favors the stabilization of ferrite particles in deionized water due to the adsorption of citrate anions onto their surface resulting in a stable and well dispersed magnetic solution [61]. These liquid suspensions were adequate to perform thermometric measurements but did not present conditions for in vitro and in vivo biomedical applications. The thermodynamic conditions of the experiment were fully modeled to obtain a direct measurement of the SLP of the magnetic powders by taking into account the heat exchange with the surrounding environment in non-adiabatic conditions and the parasitic heating of the water [60].

## 3. Results and Discussion

### 3.1. Structural Properties

**Figure 1:**
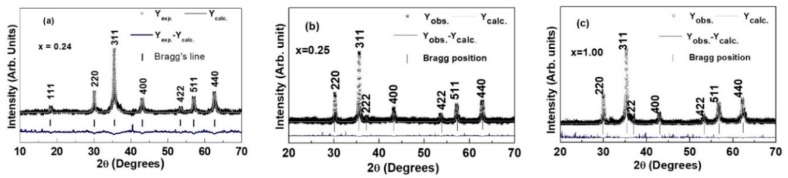
Rietveld-refined XRD patterns of: (**a**) dry gel Co_0.76_Zn_0.24_Fe_2_O_4_, (**b**) Li_0.375_Zn_0.25_Fe_2.375_O_4_ annealed at 450 °C/3 h, (**c**) ZnFe_2_O_4_ annealed at 450 °C/3 h.

Rietveld-refined X-ray diffraction (XRD) patterns of the studied samples validating the formation of the nanocrystalline mixed cubic spinel structure are presented in Figure 1. For the sample of ZnFe_2_O_4_ (Figure 1c), small traces of Fe_2_O_3_ were also observed and can be ascribed to a partial decomposition of the ferrite phase [62].

The experimental lattice parameter (*a*_exp_) values of the ferrite phases obtained from the Rietveld refinement of the XRD data are given in Table 1; the value of the Zn-ferrite sample results is higher than the ones of the LiZn-ferrite and CoZn-ferrite samples. This observed increase was due to the replacement of the Co^2+^ and Li^+^ ions characterized by an ionic radius of 0.78 and 0.70 Å, respectively, with the bigger Zn^2+^ ions (0.83 Å) [63,64,65], in agreement with Vegard’s law [66]. Moreover, the X-ray density (*ρ*_XRD_) for each sample was calculated by using the lattice parameter (*a*_exp_) and the standard formula [67]; the obtained values, ranging in the typical interval of this kind of ferrites [33,68], are given in Table 1.

The mean diameter <D_XRD_> values of the grains were obtained from the XRD data analysis by Scherrer’s equation [69] for all the samples (Table 1) and they were in the interval 32–38 nm, indicating the formation of nanocrystallites. However, the reactions used to synthesize the particles could induce a non-negligible degree of agglomeration, which could result in polycrystalline aggregates forming particles with diameters larger than <D_XRD_> [44].

These small values of <D_XRD_> revealed a significant advantage of the auto-combustion synthesis compared to the more conventional ceramic methods in order to produce ferrite particles: the lower temperature and shorter time used in the auto-combustions synthesis resulted in the grains’ smaller diameter with a greater surface area [70,71], preventing the particles from coarsening and aggregating, which is promoted by the very high temperature (T > 1000 °C) typically used in the ceramic methods.

In addition, the XRD spectra were also analyzed by the Bertaut method [55] in order to obtain the cation distribution. In particular, the cation distributions of the studied samples are given in Table 1, where the ions on the tetrahedral site (site A) are given in parentheses and the ions on the octahedral sites (sites B) between square brackets. The Zn^2+^ ions in Zn-ferrite were mainly located on the B site, whereas the CoZn-ferrite and LiZn-ferrite samples showed only a slight preference of the Zn^2+^ ions to occupy the octahedral (B) sites with respect to the tetrahedral (A) one. These observed deviations, with respect to the general preference of the Zn^2+^ ions to occupy the tetrahedral (A) site, clearly proved a non-equilibrium cation distribution in the samples. This effect was ascribed to the sol-gel auto-combustion synthesis method at a low temperature (<110 °C) and to the low efficiency of the post-synthesis heat treatment (for sample Zn-ferrite and LiZn-ferrite) which did not effectively induce a diffusion of the Zn^2+^ ions on the tetrahedral sites towards a distribution closer to the equilibrium. Instead, in the CoZn-ferrite and LiZn-ferrite samples, the divalent Co^2+^ metal ions and the divalent combination of two the metal ions [Li^+^_0.5_Fe^3+^_0.5_] [68,72] were almost totally located, as their preference, on B sites.

The cation distribution reported as the general formula $\left({\mathrm{Me}}_{\delta }^{\mathrm{II}}{\mathrm{Fe}}_{1-\delta }^{\mathrm{III}}\right)\left[{\mathrm{Me}}_{1-\delta }^{\mathrm{II}}{\mathrm{Fe}}_{1+\delta }^{\mathrm{III}}\right]O_{4}$ revealed the inversion degree of all the studied samples (δ = 0.10, 0.15, 0.09 for the CoZn, LiZn and Zn-ferrite samples, respectively), indicating an intermediate configuration of their structure between the completely inverse spinel structure (δ = 0) and the totally random distribution one (δ = 0.33).

The intermediate and tunable values of the inversion degree and its effect on the magnetic properties (see the following sections) are advantages ascribed to the auto-combustion synthesis, in which the low temperature and the fast cooling rate hinder the diffusion of the metal ions towards the equilibrium that it is typically obtained with the more conventional high-temperature ceramic methods.

### 3.2. DC-Magnetic Properties

**Figure 2:**
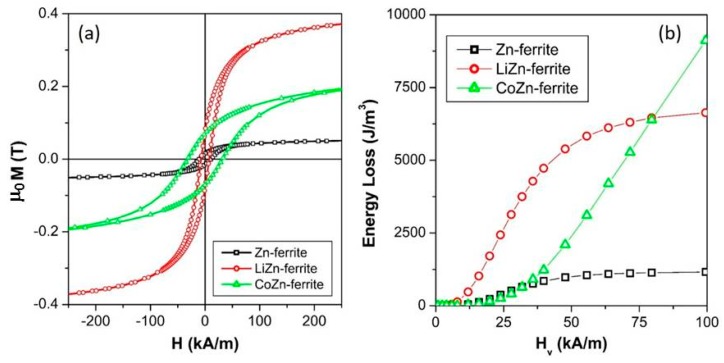
(**a**) Room-temperature major dc-hysteresis loops of all the studied samples; (**b**) dc-hysteresis loops areas as a function of the vertex field for all the studied ferrites.

An enlargement of the room-temperature dc-hysteresis loops of all the samples taken at the maximum magnetic field of 1200 kA/m is shown in Figure 2.

All curves display the typical magnetic hysteretic behavior resulting from the ferrimagnetic ordering of spinel ferrite structure [73,74], confirming the blocked state of the ferrite particles as expected by the <D_XRD_> values of the grains obtained from the XRD data analysis. The observed coercive field (*H_c_*), saturation magnetization (*μ_0_M_s_*) and the magnetic remanence (*μ_0_M_r_*) are listed in Table 2.

The complete replacement of the magnetic Co^2+^ ions with the non-magnetic Zn^2+^ ions induced a reduction of *H_c_* from 31.90 (CoZn-ferrite) to 10.65 (Zn-ferrite) kA/m. The higher value of *H_c_* of CoZn-ferrite sample was connected to the high anisotropy of Co^2+^ ions characterized by a remarkable spin-orbit coupling [36,75]. On the other hand, the replacement of the magnetic [Li^+^_0.5_Fe^3+^_0.5_] species with the non-magnetic Zn^2+^ ions promoted a slight increase of *H_c_* from 7.37 (LiZn-ferrite) to 10.65 kA/m (Zn-ferrite) and also a marked reduction of the *μ_0_M_s_* values from 0.415 T (LiZn-ferrite) to 0.066 T (Zn-ferrite). This *μ_0_M_s_* reduction can be ascribed to the different distribution of the Fe^3+^ ions in the spinel structure (see cation distribution in Table 1). In particular, in the LiZn sample, the Fe^3+^ ions were preferentially located on the octahedral sites, leading to a higher net magnetic moment than the one of the Zn sample in which a quasi-balanced distribution of Fe^3+^ ions occurred.

The experimental *μ_0_M_s_* values can be compared to the theoretical magnetization (*μ_0_M_s_^th^*) values (see Table 2) calculated in accordance to Néel’s two-sublattice model of ferrimagnets [76], where the magnetic moments of the ions (Fe^3+^ = 5 *μ*_B_, Co^2+^ = 3 *μ*_B_ and Li^+^ = Zn^2+^ = 0 *μ*_B_) on the tetrahedral and octahedral sites are considered perfectly anti-parallel, totally neglecting any temperature effect and spin disorder. The *μ_0_M_s_^th^* values for the LiZn-ferrite and CoZn-ferrite samples were very similar; therefore, the experimental *μ_0_M_s_* values are also expected to be comparable. Instead, a marked difference was measured (Table 2). This evidence clearly proves that the magnetic moments in these two ferrite samples were not perfectly antiparallel, but rather were characterized by a spin canting resulting in a non-collinear arrangement in the two-sublattices [77], especially in the CoZn-ferrite samples. The three-lattice model, suggested by Yafet and Kittel [77], confirms this hypothesis by extrapolating the canting angle values from the XRD data: 38°, 30° and 24° for the CoZn-ferrite, LiZn-ferrite, and the Zn-ferrite, respectively. Of course, the observed reduction of *μ_0_M_s_* can also be accentuated by the probable presence of a spin disorder on the ferrite surface that induced a magnetically dead layer [78,79].

The calculated values of the area (see Table 2) enclosed by the dc-hysteresis loops were proportional to the energy lost as heat by the samples in one complete major loop (hysteresis losses). It can be noted that the mixing of magnetic divalent ions (Co^2+^) or species [Li^+^_0.5_Fe^3+^_0.5_] with non-magnetic Zn^2+^ ions increased the area enclosed by the major loop with respect to the Zn-ferrite structure. This effect was particularly efficient in the CoZn-ferrite sample due to the high anisotropy of Co^2+^ ions.

Minor dc-hysteresis loops were measured for all the samples by applying selected vertex fields lower than the saturation field of the CoZn sample. The values of the area enclosed by these minor dc-loops were calculated in order to evaluate the dc-hysteresis losses as a function of selected the vertex fields (*H_v_*), see Figure 2b. All samples showed a non-linear dependence of the dc-hysteresis losses on the vertex field amplitude, which can be well described by a third-order power law in the limit of the small applied field [59]. Up to the field value of about 75 kA/m, the mutual relationship of the dc-hysteresis losses intensity of the three samples was markedly changed with respect to the dc-hysteresis losses evaluated from major dc-hysteresis loops (Table 2). In particular, in the vertex field interval 0 < *H_v_* < 75 kA/m, the LiZn-ferrite sample showed the highest hysteresis losses values whereas those of the CoZn-ferrite sample were very low because the high anisotropy of Co^2+^ ions was not completely overcome and the magnetization described narrow minor loops. When *H_v_* > 75 kA/m, the area enclosed by CoZn samples becomes the highest among the studied samples restoring the mutual relationship of the dc-hysteresis losses intensity evaluated from major dc-hysteresis loops.

### 3.3. Ac-Measurements and SLP Evaluation

Magnetic hyperthermia therapy has to comply with a variety of biological and technical constraints, among which emerges the limit for the product of magnetic field amplitude and frequency (*H* × *f*) in relation to the induced current loop diameter (D) to avoid non-specific heating in healthy areas due to Eddy currents and to avoid the stimulation of cardiac muscles and nerves [80,81]. This limit was initially proposed by Atkinson et al., as *H* × *f* < 4.85 × 10^8^ Am^−1^s^−1^ for a loop diameter of about 30 cm [27]; further experiments with a smaller diameter of the exposed body region have increased the criterion up to *H* × *f* < 5.0 × 10^9^ Am^−1^s^−1^ [28].

The range between these two criteria seems to be currently the most convenient and most used in several in vivo trials [82], at least until new studies about the biological safety of the alternating magnetic field are carried out.

In addition to this biological limitation, some engineering difficulties combined with increasing costs arise with the aim of concurrently increasing the field frequency and amplitudes in the wide space (several centimeters) required for hyperthermia treatment.

In this work, the ac-characterization was performed at the field frequency of 69 kHz and at the maximum applied magnetic field intensity, limited to 42 kA/m in order to fall within both general safety [27,28] and our technological limits. A selection of ac-hysteresis loops acquired at selected vertex fields for all samples is shown in Figure S1 of the Supplementary Materials.

**Figure 3:**
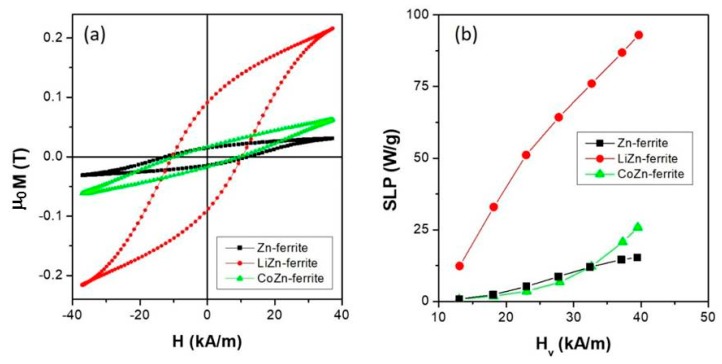
(**a**) Room-temperature minor ac-hysteresis loops of all the studied samples (f = 69 kHz and *H_v_* = 37 kA/m); (**b**) specific loss power (SLP) values for all the samples as a function of the vertex field obtained by the ac-hysteresis loops areas.

Dynamic ac-hysteresis loops (*f* = 69 kHz and *H_v_* = 37 kA/m) of all the studied powder ferrite samples are shown in Figure 3a.

In all the samples, the magnetization did not reach complete saturation, leading to minor loops. The area enclosed by the hysteresis loops represented the irreversible work dissipated in the surrounding as thermal energy profitably usable for magnetic hyperthermia [60,83,84,85].

The specific loss power is defined as the power released in the form of heat by powders submitted to electromagnetic field radiation, normalized to the mass of the solid component of the sample. In our case, the SLP was calculated from the area enclosed by the ac-hysteresis loops by the means of the following integral of the dynamic magnetization versus the applied field strength:

(2) $$SLP=\frac{f}{c}\;\oint^{\text{​}}\mu_{0}M\left(t\right)dH\left(t\right)$$

where *f* is the field frequency and *c* the weight concentration of ferrite powder. The integration was done over one period of the oscillating magnetic field.

The SLP dependence on the vertex field intensity *H_v_* of all the studied samples is shown in Figure 3b. The evaluation of this parameter represented one of the primary criteria to determine the suitability of the ferrite sample for hyperthermia applications.

In the entire investigated field range, the LiZn-ferrite sample displayed the highest SLP values indicating how the low-magnetic anisotropy divalent species [Li^+^_0.5_Fe^3+^_0.5_] favor the heat released by hysteresis losses with respect to the high-magnetic anisotropic divalent Co^2+^ ions; in particular, the SLP value of the LiZn-ferrite sample at *H_v_* = 42 kA/m is 3.6 times bigger than the one of CoZn-ferrite sample at the same vertex field. As observed from the dc-characterization reported in Figure 2b, only a further increase of *H_v_* (exceeding our technological limit) can reduce the gap between the SLP values produced by two samples leading to the promotion of the CoZn-ferrite sample. Moreover, the total replacement of the magnetic divalent ions Co^2+^ or species [Li^+^_0.5_Fe^3+^_0.5_] with non-magnetic Zn^2+^ ions hinders the heat production by hysteresis losses, resulting in low SLP values in the investigated field range with a tendency to reach a saturation value of about 15 W/g.

### 3.4. Thermometric Measurements and SLP Evaluation

**Figure 4:**
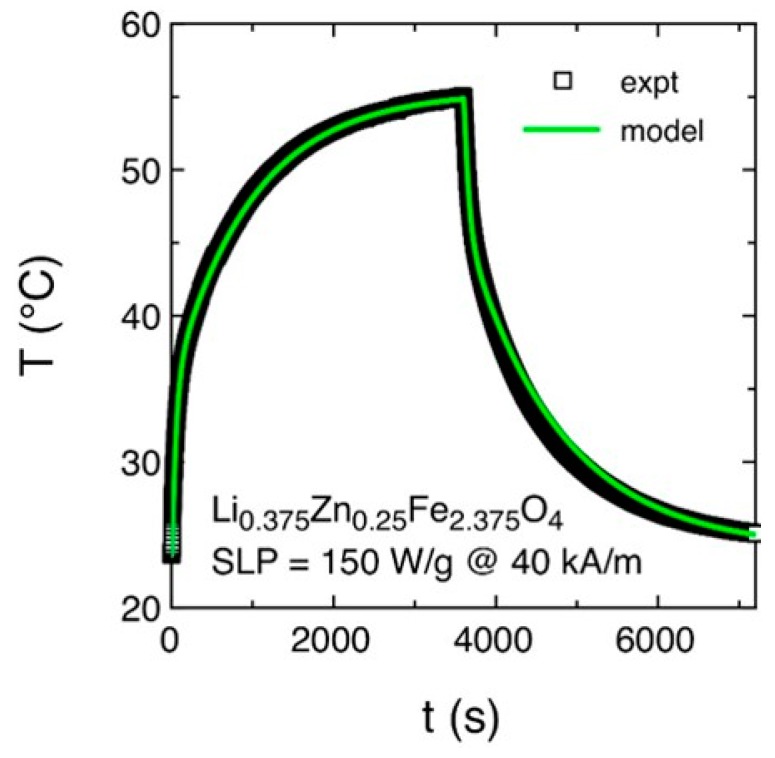
Time dependence of the temperature of the magnetic solution containing LiZn-ferrite powder under an applied field of 40 kA/m at 100 kHz. Black symbols: experimental data. Green line: best fit the by theoretical model.

Thermometric measurements were conducted in order to evaluate the ability of ferrite powders to heat the magnetic solution, in which they were dispersed, under different applied field intensities. Only the LiZn-ferrite composition was examined by thermometric measurements because it resulted in, by the ac-loops characterization, the most promising sample among those studied for hyperthermia therapy. In particular, the time dependence of the temperature of the magnetic solution containing the LiZn-ferrite powder (concentration of about 27 mg/mL) under an applied field for 40 kA/m at 100 kHz is shown in Figure 4.

The reported curve is taken as representative of all the measurements done at different applied field values from 23.9 to 47.7 kA/m. At *t* = 0 s, the radiofrequency field was switched on and the temperature of the magnetic solution increased towards the equilibrium temperature. After one hour, the radiofrequency field was switched off and the magnetic solution cooled down to room temperature. As a result, the thermometric SLP values were extrapolated by fitting the whole time evolution of the temperature of the magnetic solution by a mathematical model that takes into account the non-adiabatic condition of the exploited hyperthermia setup [60]. In Figure 4, the black symbols are the experimental data, whereas the green line is the theoretical best fit. The model based on Newton’s cooling law [86] evaluates the exchange of heat among the various components of the setup and/or the surrounding environment induced by their temperature difference. In particular, the heat released by the magnetic particles excited by an r.f electromagnetic field is all transferred to the liquid medium in which they are dispersed. Subsequently, the heat is transferred to all the experimental components and eventually to the surrounding environment. An accurate calibration procedure allows to determine the time constant of the heat exchange mechanism among the various experimental components and the other physical quantities appearing in the mathematical model. The only free parameter of the fit procedure is the power (*P*) released by the ferrite powders that it is adjusted to reproduce the whole experimental curve providing a direct estimation of the thermometric SLP of the samples:

(3) $$SLP=\frac{P}{m}$$

where *m* is the mass of ferrite powders in the liquid solution.

**Figure 5:**
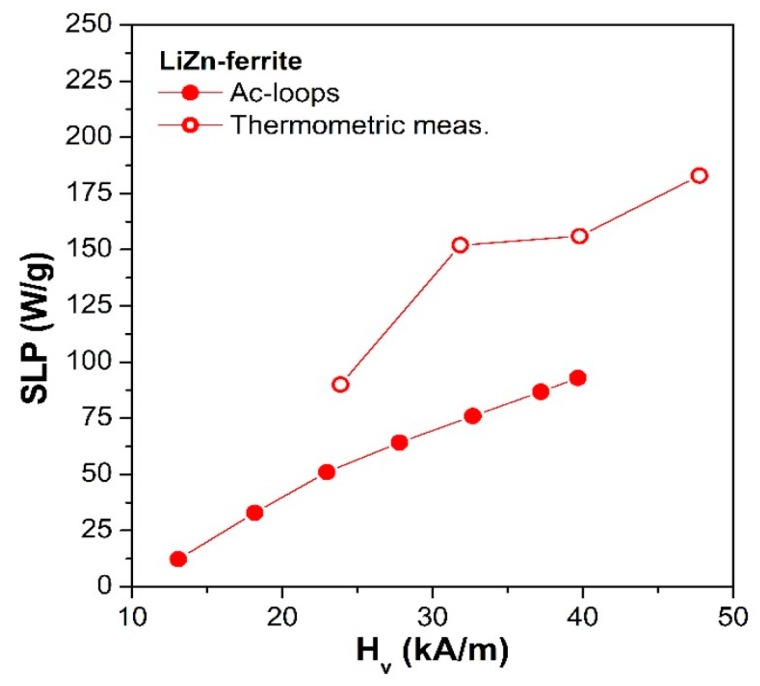
SLP values for the LiZn sample as a function of the vertex field obtained by: ac-hysteresis loops areas (full red dots) at the operation frequency of 69 kHz and the thermometric measurements (empty red dots) at the operation frequency of 100 kHz.

A summary of the SLP values determined with the two techniques for LiZn-ferrite samples is plotted in Figure 5. The SLP values calculated from thermometric measurements (empty red dots in Figure 5) turned out to be larger than the ones obtained by dynamic hysteresis loops (full red dots in Figure 5).

This effect was mainly due to the higher operating frequency of the thermometric setup (100 kHz) with respect to the B-H loop tracer (69 kHz) and it also included the contribution to the SLP of the Brown relaxation process that took place only in the magnetic liquid solution and not in the dried sample used for dynamic hysteresis loops measurements [24,87]. In fact, the Brown relaxation mechanism is associated with the physical rotation of the whole particle in the fluid generating heat due to the viscous friction between the rotating particles and the surrounding liquid medium [24,87,88]. However, recent studies, both in vivo and ex vivo, have demonstrated that the particles are generally immobilized when directly injected into the tumor tissues highlighting that the Brown process is largely suppressed during hyperthermia treatments [89,90,91]. Another significant aspect to be taken into consideration is the effect of the strong magnetic interaction that is generated by the agglomerations of particles in dried samples. In particular, the formation of agglomerates can entail an alteration of the hysteresis loop area and consequently influence the SLP value [92,93,94].

SLP values obtained for the LiZn-ferrite sample (SLP = 85–180 W/g) as reported in Figure 5 are in the same order of magnitude as the ones already reported in the literature. As examples, Mallik et al. [42] report SLP = 334 W/g (H = 33.5 mT, f = 290 kHz) for Li_0.31_Zn_0.38_Fe_2.31_O_4_ particles; Dalal et al. [95] report SLP = 143–166 W/g (H = 33.5 mT, f = 290 kHz) for Li_0.35_Zn_0.3_Co_0.05_Fe_2.3_O_4_ particles embedded in carbon nanotubes. It should be noted that the experimental results are hardly comparable as they refer to different applied fields and frequencies and strongly depend on the intrinsic properties of particles such as size, shape and composition. This comparison shows that Li-based ferrites can be considered an efficient alternative to more conventional iron-oxides such as maghemite (SLP = 106 W/g [96]) and magnetite [60] and some other ferrite compositions (SLP = 300 W/g for Gd-ferrite [97], SLP = 73 W/g for Ba-ferrite [98] and SLP = 58 W/g for Sr-ferrite [99]); however, the heat efficiency of Li-based ferrites remains much lower than Mn- and MnCo-based ferrites (SLP = 3024 W/g [100]) and magnetosomes (SLP = 960 W/g [101]).

## 4. Conclusions

Co_0.76_Zn_0.24_Fe_2_O_4,_ Li_0.375_Zn_0.25_Fe_2.375_O_4_ and ZnFe_2_O_4_ mixed-structure ferrite powders were synthesized by a sol-gel auto-combustion method. XRD spectra analysis revealed an out-of-equilibrium cations distribution ascribed to the low efficiency of the synthesis method which did not induce an effective diffusion of the Zn^2+^ ions towards the equilibrium positions. This out-of-equilibrium cation distribution strongly influenced the static and dynamic magnetic properties of the ferrites.

Dc-major loops clearly indicated that the suitable mixing of non-magnetic Zn^2+^ ions with magnetic divalent ions (Co^2+^) or species [Li^+^_0.5_Fe^3+^_0.5_] is a tool to tune the magnetic properties of the ferrite particles. In particular, the high anisotropy of Co^2+^ ions enhanced the coercive field, whereas the divalent species [Li^+^_0.5_Fe^3+^_0.5_] increased the saturation magnetization.

The magnetic energy dissipated as thermal energy profitably usable in hyperthermia was evaluated by the means of ac-hysteresis loops and thermometric measurements. Among the studied samples, the mixed LiZn-ferrite structure matches in a better way the practical requirements of heat-assisted applications than those of the mixed ferrite structure constituted by the highly anisotropic Co^2+^ ions. In the entire investigated field range, the LiZn-ferrite sample displayed the highest SLP values indicating how the low anisotropic divalent species [Li^+^_0.5_Fe^3+^_0.5_] favor the heat release by hysteresis losses with respect to the high anisotropic divalent Co^2+^ ions.

In conclusion, the SLP evaluation and the structural and magnetic characterizations of ferrite samples in dry form or dispersed in the liquid solution were calculated. This represents a preliminary and important step towards the understanding of the physical properties for their perspective use in magnetic hyperthermia and heat-assisted biomedical applications. Obviously, for prospective in vitro and in vivo studies, several other aspects should be carefully taken into account such as the size and charge of the NPs and their degree of aggregation in the magnetic solution.

## Figures and Tables

**Table 1:** Experimental lattice parameters (a_exp_); X-ray density (*ρ*_XRD_); grain mean diameter <D_XRD_> and cation distribution.

Sample	*a*_exp_ (nm)	*ρ*_XRD_ (g/cm^3^)	<D_XRD_> (nm)	Cation Distribution
Co_0.76_Zn_0.24_Fe_2_O_4_	0.8391	5.3	32	(Co_0.00_Zn_0.10_Fe_0.90_) [Co_0.76_Zn_0.14_Fe_1.10_]O_4_
Li_0.375_Zn_0.25_Fe_2.375_O_4_	0.8365	4.9	38	(Li_0.05_Zn_0.10_Fe_0.85_) [Li_0.325_Zn_0.15_Fe_1.525_]O_4_
ZnFe_2_O_4_	0.8435	5.3	35	(Zn_0.09_Fe_0.91_) [Zn_0.91_Fe_1.09_]O_4_

**Table 2:** Saturation magnetization (*μ_0_M_s_*), coercive field (*H_c_*), magnetic remanence (*μ_0_M_r_*), theoretical saturation magnetization (*μ_0_M_s_^th^*) at 0 K and the area enclosed by the major dc-hysteresis loops for all the samples.

Sample	μ_0_M_s_	H_c_	μ_0_M_r_	μ_0_M_s_^th^	Area
	(T)	(kA/m)	(T)	(T)	(J/m^3^)
Co_0.76_Zn_0.24_Fe_2_O_4_	0.255	31.90	0.071	0.52	21709
Li_0.375_Zn_0.25_Fe_2.375_O_4_	0.415	7.37	0.078	0.54	7826
ZnFe_2_O_4_	0.066	10.65	0.015	0.14	1467

## Supplementary Materials

The following are available online at https://www.mdpi.com/1424-8220/20/7/2151/s1, Figure S1: A selection of the ac-hysteresis loops acquired at the selected vertex fields for all samples.
