# Supplementary material for: Specific Loss Power of Co/Li/Zn-Mixed Ferrite Powders for Magnetic Hyperthermia
Source: Sensors (Basel). 2020 Apr 10;20(7):2151. doi: 10.3390/s20072151 (PMC7181155; doi:10.3390/s20072151)
Supplement: Supplementary file 1 [file sensors-20-02151-s001.pdf]

Supplementary Materials

Specific Loss Power of Co/Li/Zn-mixed Ferrite Powders for Magnetic Hyperthermia

Gabriele Barrera [1,*], Marco Coisson [1], Federica Celegato [1], Luca Martino [1], Priyanka Tiwari [2,3], Roshni Verma [2], Shashank N. Kane [2], Frédéric Mazaleyrat [4] and Paola Tiberto [1]

[1] Nanoscience and Materials Division, Istituto Nazionale di Ricerca Metrologica (INRiM), Strada delle Cacce 91, I-10135 Torino, Italy; m.coisson@inrim.it (M.C.); f.celegato@inrim.it (F.C.), l.martino@inrim.it (L.M.);
p.tiberto@inrim.it (P.T.)

[2] Magnetic Materials Laboratory, School of Physics, Devi Ahilya University, Khandwa road Campus, Indore 452001, India; priyanka.tiwari91092@gmail.com (P.T.); roshnikedar@gmail.com (R.V.); kane_sn@yahoo.com (S.N.K.)

[3] Department of Physics, Prestige Institute of Engineering Management and Research, Indore 452010, India

[4] Laboratory of Systems & Applications of Information & Energy Technologies (SATIE), ENS University Paris-Saclay, CNRS 8029, 61 Av. du Pdt. Wilson, F-94230 Cachan, France; Frederic.MAZALEYRAT@ens-cachan.fr

* Correspondence: g.barrera@inrim.it.

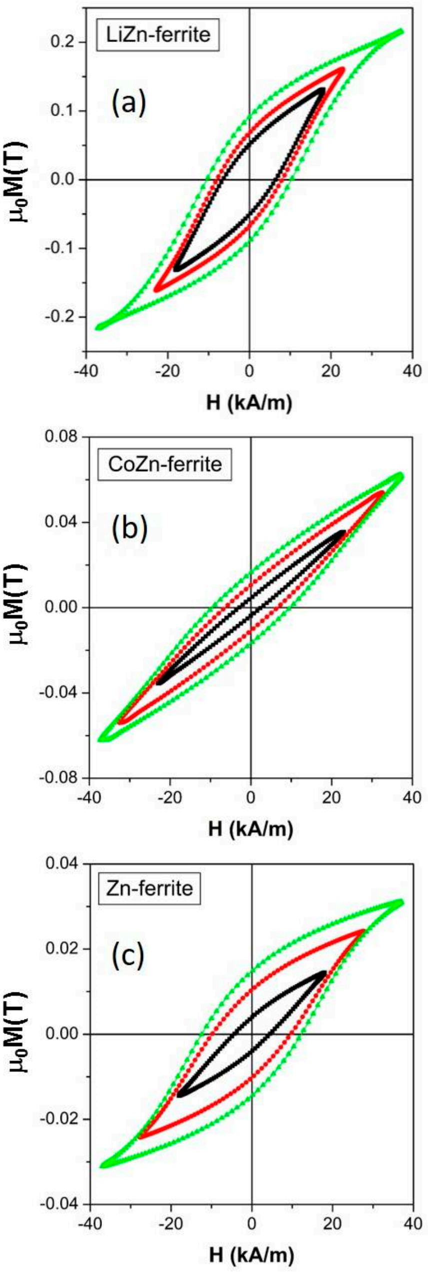

Figure S1. Room temperature minor ac-hysteresis loops (f = 69 kHz) for all studied samples: (**a**) LiZn-ferrite, (**b**) CoZn-ferrite and (**c**) Zn-ferrite.
